# Do asymptomatic STEC-long-term carriers need to be isolated or decolonized? New evidence from a community case study and concepts in favor of an individualized strategy

**DOI:** 10.3389/fpubh.2024.1364664

**Published:** 2024-04-17

**Authors:** Friedhelm Sayk, Susanne Hauswaldt, Johannes K. Knobloch, Jan Rupp, Martin Nitschke

**Affiliations:** ^1^Department of Medicine I, Division of Gastroenterology and Nephrology, University Hospital Schleswig-Holstein, Lübeck, Germany; ^2^Department of Infectious Diseases and Microbiology, University Hospital Schleswig-Holstein, Lübeck, Germany; ^3^Institute for Medical Microbiology, Virology and Hygiene, Department for Infection Prevention and Control, University Medical Center Hamburg-Eppendorf, Hamburg, Germany

**Keywords:** STEC, EHEC, socio-economic burden, social restrictions, Shigatoxin, HUS, long-term carriage, fecal shedding

## Abstract

Asymptomatic long-term carriers of Shigatoxin producing *Escherichia coli* (STEC) are regarded as potential source of STEC-transmission. The prevention of outbreaks via onward spread of STEC is a public health priority. Accordingly, health authorities are imposing far-reaching restrictions on asymptomatic STEC carriers in many countries. Various STEC strains may cause severe hemorrhagic colitis complicated by life-threatening hemolytic uremic syndrome (HUS), while many endemic strains have never been associated with HUS. Even though antibiotics are generally discouraged in acute diarrheal STEC infection, decolonization with short-course azithromycin appears effective and safe in long-term shedders of various pathogenic strains. However, most endemic STEC-strains have a low pathogenicity and would most likely neither warrant antibiotic decolonization therapy nor justify social exclusion policies. A risk-adapted individualized strategy might strongly attenuate the socio-economic burden and has recently been proposed by national health authorities in some European countries. This, however, mandates clarification of strain-specific pathogenicity, of the risk of human-to-human infection as well as scientific evidence of social restrictions. Moreover, placebo-controlled prospective interventions on efficacy and safety of, e.g., azithromycin for decolonization in asymptomatic long-term STEC-carriers are reasonable. In the present community case study, we report new observations in long-term shedding of various STEC strains and review the current evidence in favor of risk-adjusted concepts.

## Introduction

Food-borne outbreaks of severe hemorrhagic enterocolitis complicated by life-threatening hemolytic uremic syndrom (HUS) are an utterly devastating incident and a major challenge for public health (1, 2). The largest outbreak caused 3,816 documented infections in Northern Germany in 2011, including 845 cases of HUS (3). It was mediated by the Shigatoxin-producing *Escherichia coli* (STEC) strain O104:H4 and related to the consumption of contaminated sprouts. This strain harbored a phage encoding the highly pathogenic Shigatoxin type 2 (Stx 2) and expressed virulence factors of both the enterohemorrhagic (EHEC) and enteroaggregative (EAggEC) *E. coli* phenotypes. The latter might have mediated the high rate of prolonged shedding (i.e., >28 days) of viable STEC after recovery from acute diarrhea (4, 5). Infection prevention measures like sanitary separation of patients during enterohemorrhagic diarrheal disease are undisputed and essential to prevent human-to-human spread. However, the role of asymptomatic STEC carriers as a potential source of new outbreaks is controversial (5–8). Still, health authorities in most western countries are imposing far-reaching restrictions on STEC carriers. Even lenient measures like separated sanitary facilities exert psychosocial pressure and stigmatization. Ban from work, school or kindergarten might inflict substantial economic burden on the affected families and employers. Therefore, in the case of long-term STEC-shedding decolonization appears highly desirable.

Several case series suggested that antibiotic treatment for asymptomatic STEC-carriage may be an effective and safe eradication method (9, 10). In a pilot trial we previously documented that azithromycin was highly effective for the sustained decolonization of post-symptomatic long-term carriers of the highly pathogenic STEC O104:H4 outbreak strain (11). Antibiotic rehabilitation from long-term STEC carriage could stop both the risk of person-to-person transmission and set aside the social impact of exclusion policies. However, the application of antibiotics to clinically asymptomatic persons always needs appropriate justification. Moreover, antibiotic therapy is commonly discouraged in STEC-disease (12–14), since some reports have raised concerns about an inherent potential of some antibiotics to enhance Stx release and thus HUS development mainly in STEC O157.

Over the past two decades, the increasing use of syndromic multi-pathogen assays in diarrhea that detect Stx or their encoding genes has markedly increased the sporadic detection of colonization with endemic STEC strains independent of clinically overt disease or even outbreaks. This surge in detection raises questions from physicians, institutions, and public health officials about reasonable and practical measures to prevent secondary transmission. STEC is genetically a very heterogeneous and large group. The spectrum of virulence is governed in part by the subtype of Stx expressed (Stx1 or 2), and by additional pathogenicity factors, including genes encoding intestinal adherence. Different *E. coli* strains have varying pathogenic potential as proposed by the seropathotype concept (15). Moreover, the risk of long-term shedding with human-to-human transmission and, hence, the benefit from antibiotic decolonization needs strain-specific stratification. Most endemic STEC-strains have a low pathogenicity and have never been reported in the context of outbreaks or the development of HUS [for review: (10, 16–19)]. Most likely, they would neither justify antibiotic decolonization therapy nor substantiate social exclusion stipulations. Return-to-work and return-to-school polices tailored to the virulence of the STEC strain may lessen the personal and socioeconomic burden in conditions of asymptomatic long-term shedding of low-virulence organisms.

**Figure 1:**
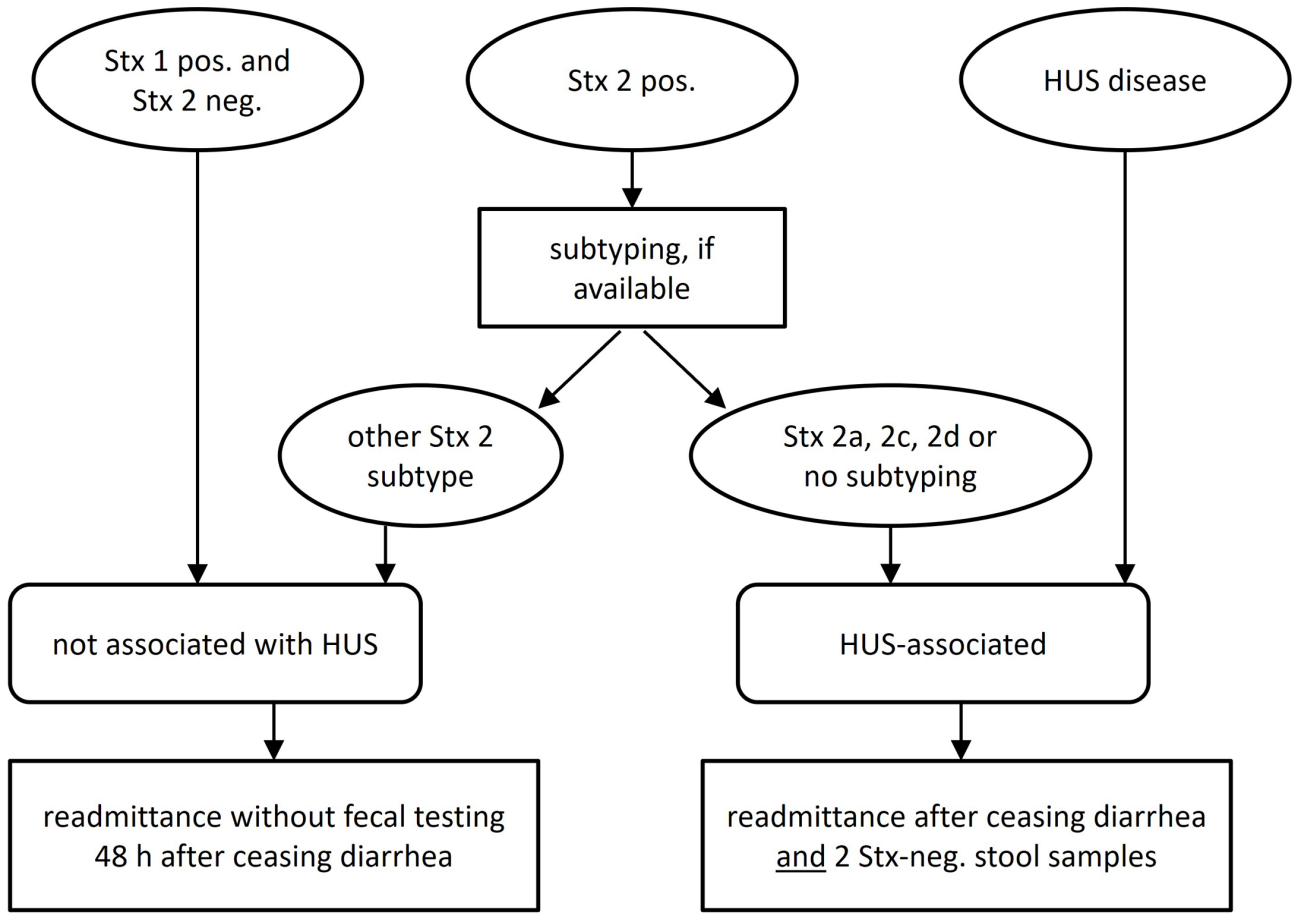
The Stx-subtypes which are HUS-associated are 2a, 2c, and 2d.

Therefore, in asymptomatic long-term STEC carriers an individualized risk-adapted approach appears mandatory. Such strategies were recently advocated by several national health authorities (Figure 1). However, the implementation in daily routine lags behind these recommendations.

## Context of the community case study–population, programmatic details and core observations

### Antibiotic eradication attempts

#### Evidence from post-symptomatic long-term carriers of the highly pathogenic outbreak-related STEC O104:H4

Following the large food-borne outbreak of STEC O104:H4 in Northern Germany in 2011 (3) a considerable number of patients showed persistent STEC-carriage (i.e., >28 days) after recovery from acute STEC-infection (4, 5). The high rate of long-term carriage was attributed to an enteroaggregative phenotype. Interestingly, STEC shedding was found to be promptly terminated by azithromycin administered as meningitis prophylaxis during off-label treatment with eculizumab in severe HUS-cases (11). Azithromycin is an approved therapy in diarrheal disease caused by enteroaggregative *E. coli* and was previously reported to reduce Stx-release *in vitro* (21, 22). Therefore, as a proof of principle, we offered a 3-day course of oral azithromycin (500 mg/d) to 15 long-term carriers (> 28 d) who - though now asymptomatic - were restricted in their social or working life. After the 3-day course all had consistently negative stools without any HUS related symptoms (11). This observation required approval in a greater cohort of long-term carriers of STEC O104:H4.

Accordingly, we treated 27 additional cases, totaling *n* = 42 long-term carriers with a history of acute STEC-enterocolitis and/or HUS. They all had completely recovered but – though being asymptomatic – showed persistent fecal STEC shedding beyond day 28 from the onset of diarrheal symptoms. Patients were referred to our outpatient clinic due to their individual burden of social and economic restrictions.

**Figure 2:**
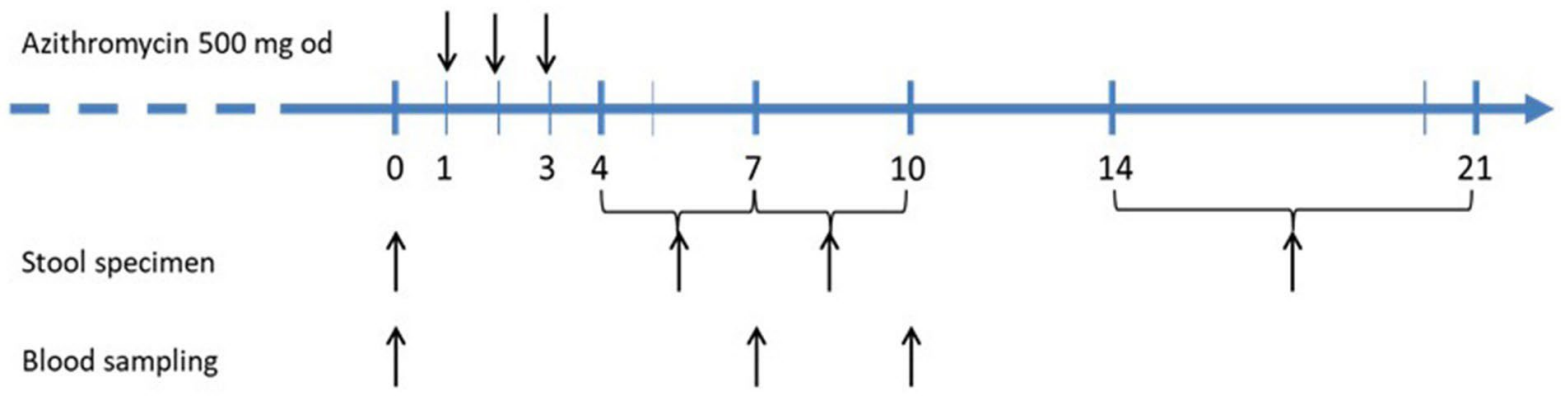
Treatment protocol for decolonization of STEC-long-term carriers with short course azithromycin.

The decolonization protocol is visualized in Figure 2. Details on microbiologic analysis are presented in the online supplement. Persistent STEC shedding was documented within the last 7 days prior to the decolonization attempt. The core efficiency parameter was the rate of sustained microbiological response vs. rate of relapse/persistence at 2–3 weeks after azithromycin treatment. The safety outcome comprised any clinical or laboratory signs of HUS and/or any other clinical adverse event.

The median duration from the onset of outbreak-related diarrheal symptoms to the start of decolonization therapy was 60 days (range 30–189, mean duration73.5 ± 39.4 days) in 39 of 42 persons of this cohort. The remaining three individuals, however, did not report a preceding diarrheal episode, but were eventually detected. E.g., one of them was found positive at screening as a household-contact about 10 days after his wife had developed STEC-diarrhea. It is unclear whether he had the same nutritional source of infection as his wife or had acquired a secondary person-to-person infection.

**Figure 3:**
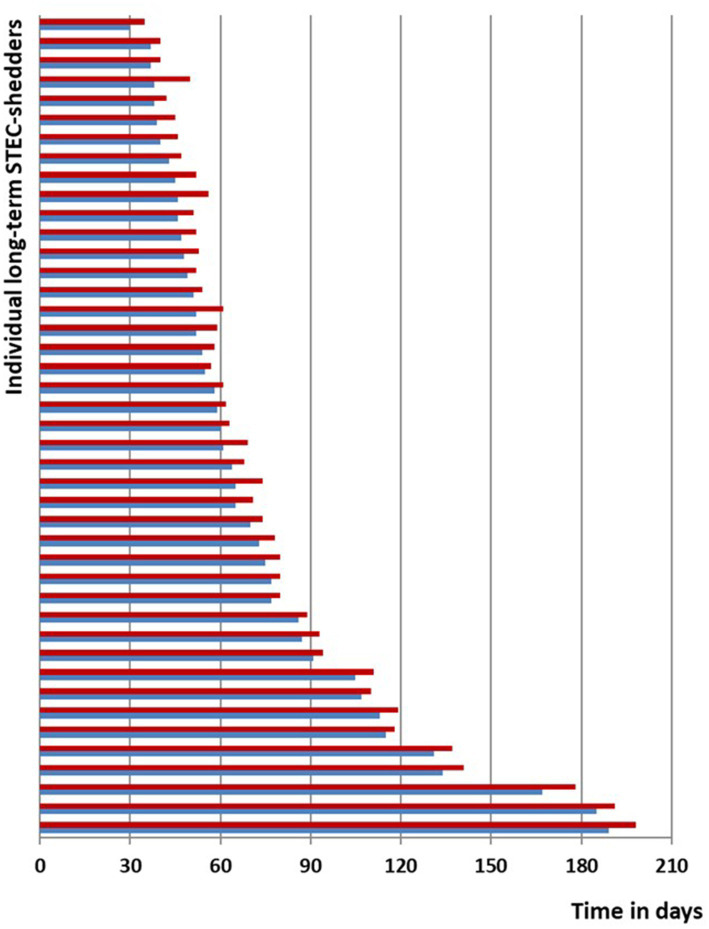
Line-list STEC O104:H4 cases; blue, time span of documented STEC shedding since symptom onset or first diagnosis until start of decolonization with azithromycin; red, time span until negative stool tests.

41 (98%) participants were successfully decolonized as confirmed by at least 3 negative stool samples within the subsequent 21 days. The median duration from acute symptom onset until first negative stool following the 3-day azithromycin course was 63 days (range 35–198). However, 1 person showed a relapse/recurrence of STEC-positivity after two samples had been negative. In this individual a second 3-day course of azithromycin was repeated after another 7 weeks of positive stool samples and then lead to prompt and sustained decolonization (Figure 3). None of the subjects demonstrated any HUS-related clinical or laboratory deterioration. There were no adverse events apart from abdominal discomfort in two participants that lasted less than 2 days while continuing azithromycin.

In this cohort of O104:H4 long-term carriers, individuals judged that their expected personal benefits from decolonization would far exceed the potential risk of adverse events. Some of them were at risk of losing their jobs after prolonged ban from work. One individual, e.g., was blocked from running his restaurant for weeks, another was suspended from his food-processing job for almost 3 months, one adolescent patient was not allowed to travel abroad as an au-pair and one child was banned from kindergarten for several weeks, which severely restrained its parents’ working life. One married couple first declined a decolonization attempt and then stayed at home for about 6 months awaiting spontaneous decolonization. As shown in the line-list (Figure 3), they continued to be positive after 180 days, but even then, therapy with azithromycin promptly led to sustained decolonization.

#### Community case series of sporadic asymptomatic long-term carriers of endemic strains

Additionally, we collected sporadic community cases colonized with endemic non-O104:H4, non-O157 STEC over 10 years (2012–2021). All were incidentally detected, and they had no history of gastrointestinal disease or HUS, and none was associated with an outbreak. Therefore, the duration of shedding was undetermined, but all of these subjects had repeatedly been found STEC-positive since ≥28 days. They reported severe social restrictions inflicted by local public health authorities for several weeks or months. Nationwide, we encountered about 50 contacts for counseling initiated either by the colonized individuals, by their general practitioner or via public health office seeking advice for decolonization. We carefully discussed the individual pros and cons to perform a decolonization attempt with azithromycin. Some decided not to undergo a decolonization attempt. In other cases, information on the strain-serotype or the subtyping of Stx-1 vs. 2 were incomplete as such diagnostic workups are regularly not reimbursed by health insurance coverage. Therefore, we here report 21 sporadic asymptomatic long-term STEC-carriers with confirmed endemic non-O104 strains treated with azithromycin. Of these, 10 carried serotype O91:H14, a subtype with H14-flaggelin which has never been associated with STEC-outbreaks or HUS (23). During the last two decades the fraction of O91 strains has significantly increased, according to the German National Reference Centre for Enteric Bacterial Pathogens run by the Robert-Koch-Institute (RKI) (16). In 4 subjects we found serogroup O26, 2 harbored O113, and one was found positive for O15, O76, O146, O156, or O181, respectively. Patients decided that their individual burden of exclusion outweighed any potential risk of decolonization treatment. They took full responsibility for their decision to undergo the off-label decolonization attempt according to the above-mentioned protocol, and to perform safety checks. Decolonization was successful in all 21 individuals as determined by the family physician’s report of negative follow-up stool samples. None of them reported any signs of HUS or other significant adverse events.

## Discussion

### Pros and cons of antibiotic therapy in STEC-disease and long-term shedding

Evidently, azithromycin is highly effective for the sustained decolonization of asymptomatic long-term STEC O104:H4 carriers as well as asymptomatic long-term shedders of endemic STEC strains. This approach appeared safe, since no HUS-related clinical or laboratory deterioration occurred. In contrast to these findings in asymptomatic long-term carriers, antibiotics are generally discouraged during STEC-related acute bloody diarrhea (12–14). *In vitro*, Stx production is boosted by sub-inhibitory concentrations of specific antibiotics. Data are available mainly for EHEC O157 and for two classes of antibiotics, the fluoroquinolones and trimethoprim-sulfamethoxazol (TMP/SMZ), which have repeatedly been shown to induce Stx production *in vitro*. This is plausible, as both antibiotics, targeting DNA-synthesis, induce the bacterial SOS stress response to DNA damage, which is linked to an increase in phage production and toxin release (24–26). Additionally, a retrospective analysis from the US FoodNet surveillance recently reported an augmented risk of HUS among children and adults with O157 diarrhea treated with β-lactams (14). For other strains, data were conflicting and hardly comparable due to different antibiotics at variable doses and variable susceptibility profiles. Currently, there is no rational to suggest alternative antibiotics without prior testing of antimicrobial resistance. Harm from antibiotic treatment has never been proven through randomized controlled trials, and observational studies suffer from biases such as greater likelihood of antibiotic treatment in patients presenting with more severe illness. During the O104-outbreak in 2011 the use of several antibiotics for concurrent reasons did not deteriorate clinical outcome according to an observational multicenter study (27).

Azithromycin, an antibiotic of the macrolide family, binds to the 50S subunit of the bacterial ribosome. Azithromycin inhibits protein synthesis including the production and release of Shiga toxin *in vitro* independent from its bacteriostatic effects (21, 22, 28). In addition, azithromycin has modulating effects on the Stx-induced inflammatory reaction on the vascular endothelium. Whether azithromycin could reduce the duration of diarrhea or protect against the development of HUS in highly pathogenic STEC is an unsolved question. Currently, there is no evidence that antibiotic treatment is harmful once HUS has developed. Studies on animal models showed a drastic reduction in HUS-related mortality. Nonetheless, we have to await the results of an ongoing clinical trial (ClinicalTrials.gov number, NCT02336516) of azithromycin therapy initiated during established HUS.

Moreover, we found no report on HUS-induction in asymptomatic long-term STEC-carriers receiving antibiotics for concurrent indications (10). Based on a recent meta-analysis including 10 clinical and 22 *in vitro* studies, as well as data from the Danish cohort of registered STEC infections, antibiotic treatment with protein and cell-wall synthesis inhibitors can be considered safe in chronic STEC-carriers when specific criteria regarding patient group, serotype and virulence profile are met (18, 29). Case reports suggested that antibiotic treatment for asymptomatic STEC-carriage may be a safe eradication method for less virulent strains (9, 10). This is confirmed by our present collection of anecdotic endemic cases.

In general, the advantages of antibiotic treatment must always be balanced against the disadvantages of short and long-term interference with the human intestinal microbiome. It is important to consider that the dissemination of plasmide-borne macrolide resistance may reduce the potential benefit of azithromycin for STEC decolonization, and determination of azithromycin MICs should be considered (30). Other treatment options especially in acute STEC disease, including Stx-receptor analogs, antibodies against LPS, use of probiotics as well as phages and vaccines, have been reviewed elsewhere (31, 32). In brief, they did not provide novel successful concepts – neither for acute disease nor in asymptomatic long-term carriage.

### Human-to-human transmission

The infectious potential of asymptomatic STEC shedding for human-to-human transmission is unclear. Present knowledge about person-to-person STEC-transmission is predominantly deduced from serogroup O157, a group that mainly affects young children. In childcare settings, secondary cases via human-to-human spread during active diarrhea are frequent and this is advocated as an important mechanism during outbreaks (33). During the acute diarrheal phase persons appear to be more likely to spread STEC than asymptomatic long-term shedders (6, 7). In registries of STEC-O157 cases from England (*n* = 225 children <6 yrs), Scotland (*n* = 2.228 cases over 10 years) and the US secondary cases due to fecal-oral transmission occurred in 10–14% of all cases with confirmed acute enterocolitis. The mean time between onset in primary and secondary cases was about 8 days (range 3–24 days); pathogen transmission from asymptomatic O157-carriers was not observed (33–35). During the O104:H4 outbreak in 2011 only few cases of secondary human-to-human household transmission were reported. Most of them occurred early, i.e., during the acute diarrheal phase of the primary case (5); the risk of transmission from asymptomatic long-term STEC shedders appeared much lower. In a prospective post-outbreak surveillance in 2011 run by German health authorities in order to detect further infections after the outbreak’s end, 33 post-outbreak cases were recorded based on mandatory reporting from summer until the end of 2011. These post-outbreak cases occurred with decreasing frequency over the 6 months follow-up period and were clinically rather mild. Most of them had previous contact with known outbreak cases or were mediated by laboratory or nosocomial spread but were not related to sprout consumption (36). Evidently, the pathogenic outbreak strain STEC O104:H4 has the potential to prolong chains of human transmission, with long-term shedding being the most relevant risk factor. Still the number of secondary cases was low (< 1%) compared to the food-borne cases (*n* = 3.816). For endemic non-pathogenic STEC-strains there are no valid data on person-to-person transmission.

### Strain specific pathogenicity factors for HUS-development and long-term shedding

Shiga-toxins and the adherence factor intimin (eae) /enterocyte effacement pathogenicity island are the main virulence factors of STEC. They cause the attaching and effacing lesions on infected epithelial cells. Moreover, expression of phage-encoded Stx-subtype 2a, 2c or 2d appears to be responsible for the intestinal vascular damage that characterizes STEC-mediated hemorrhagic enterocolitis and to induce the systemic complications like hemolysis, renal failure and neurologic deterioration seen in HUS. The O104:H4 outbreak strain harbored a phage encoding Stx2a, which is associated with the high rate of HUS observed. Stx-1 expression, in contrast, is in general not associated with HUS. Genomic plasticity and horizontal gene transfer enable the emergence of STEC strains with additionally acquired virulence properties. The Stx gene distribution changed, for example, from only 7% of STEC O26:H11 harboring Stx2 in 1999 to 59% in 2013 (37). Therefore, it is not possible to predict the emergence of „new‟ highly pathogenic STEC types based solely on the presence of any unspecified Stx or by focusing on a restricted panel of serogroups. Next to molecular strain-typing, whole-genome sequencing, including differentiation of Stx1 and 2 allelic variants, might help to assess the risk inherent to long-term STEC carriage. Further molecular details have been discussed elsewhere (15–19, 38).

Differential bacterial mechanisms of intestinal adherence and host factors may result in variable shedding dynamics of diverse STEC-strains. In some individuals long-term STEC carriage was documented for up to 1 year. STEC O104:H4, the outbreak strain in Germany in 2011 (3) expressed virulence factors of enteroaggregative (EAggEC) phenotype attributing to a high rate of persistent STEC-carriage (i.e., >28 days) after recovery from acute STEC-infection (4, 5). STEC O157 – another highly pathogenic serogroup which is primarily responsible for outbreaks and severe HUS in children – in contrast, rarely persists for >28 days (39). For endemic STEC which are incidentally detected in asymptomatic carriers without antecedent diarrheal disease or HUS, the period of shedding is unknown.

### Pros and cons of social restriction policies

In many countries worldwide far-reaching restrictions are enunciated by health authorities for those asymptomatic STEC carriers who might constitute a potential risk of infecting other persons or contaminating food items irrespective of the strain. This sounds justified for highly virulent strains shed by persons in food-sensible context. However, in cases of prolonged shedding, social restrictions beyond personal hygiene can be onerous. Exclusion of asymptomatic persons from normal daily life with the risk of losing their jobs imposes a severe economic burden in addition to psychosocial pressure and stigmatization. Given the increased detection rate of endemic STEC, risk-adjusted modifications appear mandatory. In Denmark, subtyping of Stx is routinely integrated into public health strategies since 2015 in order to focus follow-up surveillance to patients infected with high-risk strains (18). Recently, in Norway and Germany the official rules for re-admittance of STEC-carriers to work, to school or kindergarten were greatly revised by the legal health authorities (20, 40). The new recommendations adhere to a strain-specific stratification. In Germany, patients infected with STEC expressing Stx-1 and sporadic cases of asymptomatic colonization with endemic strains, e.g., do not need any further fecal controls and should no longer suffer from restricted daily social interaction (Figure 1). Syndromic PCR panels that do not differentiate between Stx1 and 2, however, provide inadequate information. In our collection of endemic STEC long-term carriers, subjects reported ongoing exclusion policies even though their subtyping indicated no substantial pathogenic threat, mostly before 2020. According to the new national standards, most of them would retrospectively not require any precautionary restraints and therefore would no longer apply for eradication with azithromycin today.

Epidemiologically, all large STEC-outbreaks over the last 50 years were food-borne. Next to contaminated vegetable, products of domestic and wild animals served as STEC-vehicles. Ruminants, especially cattle, are regarded as important sources of food-borne STEC-transmission to humans. STEC strains persisting in cattle for longer periods can serve as gene reservoirs that supply *E. coli* with virulence factors, thereby generating new potential outbreak strains. Moreover, there are multiple reports from all over the world showing a considerable prevalence of plasmid-borne antimicrobial (multidrug) resistance of various STEC strains in domestic and even in hunted wildlife animals. This clearly precludes benefit from the broad use of antibiotics to prevent outbreaks. Given the globalization of food chains and human travel, resistance patterns in far distant regions might well have relevance on public health elsewhere, nowadays. The O104:H4 outbreak in 2011, e.g., was mediated by contaminated sprouts imported from overseas, and this strain was resistant to ß-lactams (ESBL), tetracycline, streptomycin and trimethoprim/sulfamethoxazole. In contrast, there are no reports on outbreaks that were triggered from asymptomatic human long-term STEC carriers. To the best of our literature search, there is no epidemiologic evidence of any public health benefit from social exclusion policies for asymptomatic long-term STEC-carriers. Public health attempts to reduce the human risk for acquiring STEC infections should therefore mainly address strategies to control persisting STEC strains in the food-chain. Animal carriage of STEC is reduced, e.g., through vaccination and improved farm practices (41). To decently review the large body of scientific literature concerning this highly relevant veterinary issue is far beyond the scope of this article. In summary, preventing STEC transmission from animals and nutritional environments to humans includes appropriate food preparation, personal hand hygiene, control of environmental contamination, and food and water quality. This might be much more effective than the social exclusion of asymptomatic human carriers of low-virulent strains (42).

### Limitations

The conclusions drawn from this community case study are inevitably subject to some limitations. The German O104-STEC-outbreak in 2011 was one of the largest worldwide, and likewise, our cohort of long-term-carriers decolonized with azithromycin is the largest reported. Still the number of cases in our decolonization cohorts is low to conclude strict recommendations. To overcome the substantial lack of scientific evidence, central registries are needed. They might aim at systematically determining strain-specific risks of human-to-human transmission in asymptomatic long-term carriers as well as benefit vs. harm from social restriction. Finally, antibiotic decolonization approaches need confirmation in prospective controlled studies. This includes a more in depth specification of the optimum follow-up period after decolonization to rule-out relapses.

## Recommendation for an individualized risk-adjusted strategy

The above issues endorse the recent concept of an individualized approach that takes strain-specific risks and personal and public threats into account. Molecular strain-profiling in long-term STEC carriage would trigger stringent hygiene measures reserved to high-risk strains and limit unnecessary precautionary measures in low-virulent STEC. This, however, mandates molecular microbiologic diagnostics beyond the routine of simply detecting Stx by ELISA or by PCR (Figure 1). This work-up is still not regularly reimbursed by health insurance policies. On a public health level, such molecular stratification, however, might well be cost-effective.

From a clinical point of view, the previous dogma that antibiotics are absolutely contraindicated in STEC infection needs to be revised. To date, antibiotics should be handled cautiously in patients with acute bloody diarrhea caused by STEC, especially if caused by STEC O157. In long-term shedding of highly virulent STEC, decolonization by a short course of oral azithromycin might offer an appropriate option. Our community case study confirms that a 3-day course is highly effective and safe. Decolonization with azithromycin could shorten the duration of human STEC shedding, and thereby reduce the risk of transmission and the need of prolonged restrictions. The potential benefit is underlined by the evidence of some human-to-human post-outbreak transmissions during the 6 months of national surveillance after the O104:H4 outbreak in 2011 (36). Asymptomatic carriers of endemic non-virulent STEC strains, in contrast neither need prolonged restrictions, nor fecal follow-up testing, and therefore, do not require antibiotic decolonization treatment (20). Strain-specific risk stratification allows for a risk-adjusted individual strategy. Together with our eradication approach reserved to high-risk pathogens, this could modify public health surveillance, enable an earlier return to normal life for many long-term carriers, and hence reduce the individual and socioeconomic burden of long-term STEC-carriage.

## Data availability statement

The raw data supporting the conclusions of this article will be made available by the authors, without undue reservation.

## Ethics statement

Ethical approval was not required for the studies involving humans because community case study, part of it was interventional (with ethical approval), part of it was rather observational (not approved). The studies were conducted in accordance with the local legislation and institutional requirements. The participants provided their written informed consent to participate in this study.

## Author contributions

FS: Conceptualization, Data curation, Formal analysis, Investigation, Methodology, Project administration, Resources, Supervision, Validation, Visualization, Writing – original draft, Writing – review & editing. SH: Data curation, Investigation, Methodology, Resources, Validation, Writing – review & editing, Conceptualization, Project administration, Writing – original draft. JK: Conceptualization, Data curation, Formal analysis, Investigation, Methodology, Resources, Supervision, Validation, Writing – review & editing. JR: Conceptualization, Resources, Supervision, Writing – review & editing. MN: Conceptualization, Data curation, Formal analysis, Investigation, Methodology, Resources, Supervision, Validation, Writing – review & editing.
