# Correction: Do asymptomatic STEC-long-term carriers need to be isolated or decolonized? New evidence from a community case study and concepts in favor of an individualized strategy

**DOI:** 10.3389/fpubh.2026.1802480

**Published:** 2026-03-13

**Authors:** Friedhelm Sayk, Susanne Hauswaldt, Johannes K. Knobloch, Jan Rupp, Martin Nitschke

**Affiliations:** 1 Department of Medicine I, Division of Gastroenterology and Nephrology, University Hospital Schleswig-Holstein, Lübeck, Germany; 2 Department of Infectious Diseases and Microbiology, University Hospital Schleswig-Holstein, Lübeck, Germany; 3 Institute for Medical Microbiology, Virology and Hygiene, Department for Infection Prevention and Control, University Medical Center Hamburg-Eppendorf, Hamburg, Germany

**Keywords:** STEC, EHEC, socio-economic burden, social restrictions, Shigatoxin, HUS, long-term carriage, fecal shedding

There was a mistake in the caption of Figure 1 as published. The corrected Figure 1 and the caption appear below.

**Figure 1:**
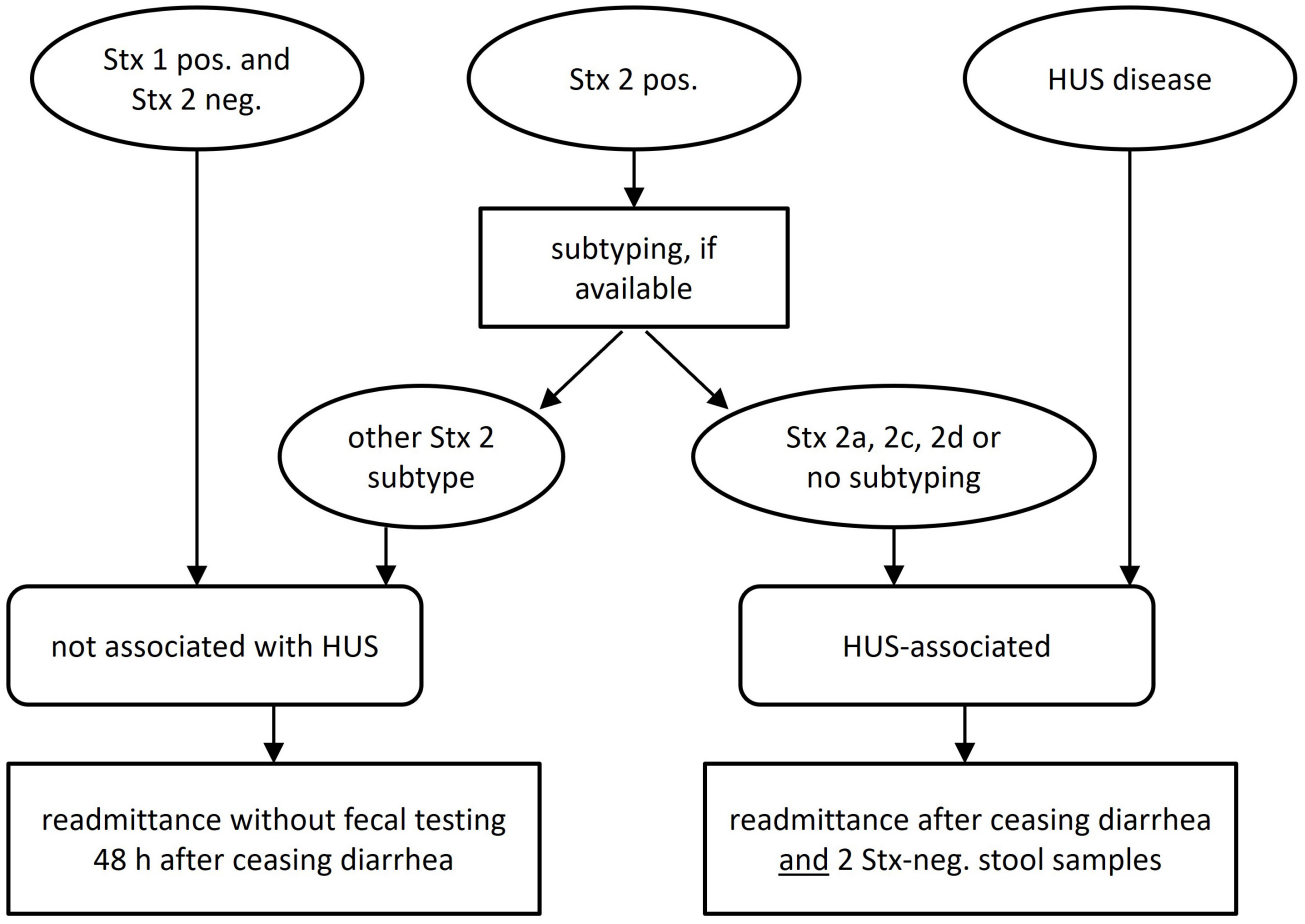
The Stx-subtypes which are HUS-associated are 2a, 2c, and 2d.

Figure 1. The Stx-subtypes which are HUS-associated are 2a, 2c, and 2d.

The reference for 36 was erroneously written as “Frank, **D**, Milde-Busch, A, and Werber, D. Results of surveillance for infections with Shiga toxin-producing Escherichia coli (STEC) of serotype O104:H4 after the large outbreak in Germany, July to December 2011. *Euro Surveill*. (2014) 19:20760. doi: 10.2807/1560-7917.es2014.19.14.20760”. It should be “Frank **C**, Milde-Busch A, Werber D. Results of surveillance for infections with Shiga toxin-producing Escherichia coli (STEC) of serotype O104:H4 after the large outbreak in Germany, July to December 2011. *Euro Surveill*. (2014) 19:20760. doi: 10.2807/1560-7917.es2014.19.14.20760”.

The email ID of corresponding author was erroneously given as “friedhelm.sayk@uksh.de”. The correct mail ID is “f.sayk@drk-krankenhaus.de.”

The original version of this article has been updated.

