# Supplementary material for: Do asymptomatic STEC-long-term carriers need to be isolated or decolonized? New evidence from a community case study and concepts in favor of an individualized strategy
Source: Front Public Health. 2024 Apr 17;12:1364664. doi: 10.3389/fpubh.2024.1364664 (PMC11064650; doi:10.3389/fpubh.2024.1364664)
Supplement: Supplementary file 1 [file Data_Sheet_1.PDF]

Electronic supplemental material

Details on Microbiology

For the cultivation of STEC strains from PCR-confirmed stx-positive stool samples from affected individuals, three-loop smears were prepared on MacConkey Agar (MCK, bioMérieux, Marcy l'Etoile, France) and STEC-CHROMagar™ (CHROMagar, Paris, France) and incubated at 42 °C or 37 °C, respectively. In the absence of growth on STEC-CHROMagar™, at least 10 single colonies were isolated as pure cultures and examined for the presence of stx1 or stx2 by PCR. Serotypes of successfully isolated STEC were determined by gnd typing (Gilmour et al. 2007, https://doi.org/10.1099/jmm.0.47053-0). Briefly, DNA from strains isolated in pure culture was obtained either by heat extraction (20 min at 99° C) in 200 µl in RALF buffer (AmplexDiagnostics GmbH, Gars-Bahnhof, Germany), by automated extraction using the NucliSens® easyMag® (bioMérieux) system or by manual extraction using the UltraClean® Microbial DNA Isolation Kit (Mo Bio Laboratories, Carlsberg, CA USA). 2 µl of the obtained DNA solution was used as template in a PCR with the primers gnd_f (GGCTTTAACTTCATCGGTAC) and gnd_r (TCGCCGTAGTTCAGATCCCA) and a Taq polymerase (Thermo Fisher Scientific, Waltham, MA USA) and amplified on a FlexCycler thermal cycler (Analytik Jena AG, Jena, Germany). The PCR reaction products were separated using agarose gel. Bands of the respective strains with the expected amplicon size were purified from the gels using NucleoSpin® PCR Clean-Up Kits (Macherey-Nagel GmbH, Düren, Germany) and subsequently sequenced by an external service provider (Eurofins Genomics MWG, Ebersberg, Germany). The gnd sequences were corrected, trimmed to the defined length and checked using Vector NTI 11.5 software (Thermo Fisher Scientific). The serotypes were determined on the basis of the gnd sequences using the E. coli O-typer (Gilmour et al. 2007) by comparison with the gnd reference sequences stored there.

Decolonization protocol (Figure 2)

Prior to azithromycin treatment, candidates were asked to provide stool specimen for updated cultural confirmation of ongoing STEC-shedding and serotyping of the respective strain. Positive cases received 500 mg qd of azithromycin for 3 days (day 1-3), for children dosage was body-weight adapted (10 mg/kg). Patients did not take any concurrent antibiotic during the eradication protocol.

They attended three visits at the outpatient clinic – the first prior to decolonization treatment (day 0), then twice after completing the azithromycin course (days 7 and 10). At each visit patients were monitored for symptoms or clinical signs suggestive of HUS or relevant side effects. Blood was drawn at each visit in order to exclude HUS-related laboratory changes (thrombocytes, hemoglobin, serum-Creatinine, LDH and bilirubin). Following the full 3-day azithromycin course 3 stool samples were collected: (1) between day 4-7, (2) between day 7-10, and (3) at about day (14-)21.

Data handling and statistics

Long-term carriage in our outbreak-related O104:H4-cohort was defined as confirmed shedding > 28 days. Decolonization success in this cohort was analyzed by line-listing of microbiologic stool results from the onset of outbreak-related diarrheal symptoms until sustained microbiological response. The decolonization response rate vs. rate of failure or relapse, and the occurrence of adverse events were calculated and reported descriptively.

STEC-cohort	O104:H4	endemic non-O104, non-O157
number of cases reported	n=42	n=21
successful decolonization, confirmed 2-3 weeks after azithromycin	all	all<br>*according to family physician report
Number of decolonization failure or STEC-relapse	1<br>*stable successful decolonization after second 3-day course of azithromycin	none<br>*according to family physician report
Duration of confirmed carriage<br>*since start of outbreak related diarrheal episode before start of azithromycin (days)	median 60 (range 30-189), mean 73.5 (±39.4 SD)	> 28 days<br>*cases sporadically detected without history of antecedent diarrheal episode, therefore time of intestinal infection and duration of shedding period unknown
Duration of confirmed carriage since start of outbreak related diarrheal episode until first negative stool samples following azithromycin (days)	median 63 (range 35-198)	promptly negative stool samples<br>*according to family physician report
HUS-development following decolonization treatment	none	none
other severe adverse events	none	none<br>*according to family physician report

Supplemental table legend

Efficacy and safety of decolonization with azithromycin in long-term carriers of outbreak-related pathogenic STEC O104:H4 and community cases of endemic non-O104, non-O157 STEC strains.
